# Clinical and Virological Study of Dengue Cases and the Members of Their Households: The Multinational DENFRAME Project

**DOI:** 10.1371/journal.pntd.0001482

**Published:** 2012-01-24

**Authors:** Philippe Dussart, Laurence Baril, Laure Petit, Lydie Beniguel, Luong Chan Quang, Sowath Ly, Raimunda do Socorro Silva Azevedo, Jean-Baptiste Meynard, Sirenda Vong, Loïc Chartier, Aba Diop, Ong Sivuth, Veasna Duong, Cao Minh Thang, Michael Jacobs, Anavaj Sakuntabhai, Marcio Roberto Teixeira Nunes, Vu Ti Que Huong, Philippe Buchy, Pedro Fernando da Costa Vasconcelos

**Affiliations:** 1 Institut Pasteur de la Guyane, Laboratoire de Virologie, Cayenne, French Guiana; 2 Institut Pasteur, Unité d'Epidémiologie des Maladies Emergentes, Paris, France; 3 Institut Pasteur de Dakar, Unité d'Epidémiologie des Maladies Infectieuses, Dakar, Senegal; 4 Institut Pasteur d'Ho Chi Minh Ville, Unité d'Epidémiologie, Ho Chi Minh City, Vietnam; 5 Institut Pasteur du Cambodge, Unité d'Epidémiologie et de Santé Publique, Phnom Penh, Cambodia; 6 Instituto Evandro Chagas, Department of Arbovirology and Hemorrhagic Fevers, Belém, Brazil; 7 Institut Pasteur de la Guyane, Unité d'Epidémiologie, Cayenne, French Guiana; 8 Institut Pasteur du Cambodge, Unité de Virologie, Phnom Penh, Cambodia; 9 Institut Pasteur de Ho Chi Minh Ville, Laboratoire des Arbovirus, Ho Chi Minh City, Vietnam; 10 University College London Medical School, Department of Infection, London, United Kingdom; 11 Institut Pasteur, Unité de Pathogénie Virale, Paris, France; University of California, Berkeley, United States of America

## Abstract

**Background:**

Dengue has emerged as the most important vector-borne viral disease in tropical areas. Evaluations of the burden and severity of dengue disease have been hindered by the frequent lack of laboratory confirmation and strong selection bias toward more severe cases.

**Methodology:**

A multinational, prospective clinical study was carried out in South-East Asia (SEA) and Latin America (LA), to ascertain the proportion of inapparent dengue infections in households of febrile dengue cases, and to compare clinical data and biological markers from subjects with various dengue disease patterns. Dengue infection was laboratory-confirmed during the acute phase, by virus isolation and detection of the genome. The four participating reference laboratories used standardized methods.

**Principal Findings:**

Among 215 febrile dengue subjects—114 in SEA and 101 in LA—28 (13.0%) were diagnosed with severe dengue (from SEA only) using the WHO definition. Household investigations were carried out for 177 febrile subjects. Among household members at the time of the first home visit, 39 acute dengue infections were detected of which 29 were inapparent. A further 62 dengue cases were classified at early convalescent phase. Therefore, 101 dengue infections were found among the 408 household members. Adding these together with the 177 Dengue Index Cases, the overall proportion of dengue infections among the study participants was estimated at 47.5% (278/585; 95% CI 43.5–51.6). Lymphocyte counts and detection of the NS1 antigen differed significantly between inapparent and symptomatic dengue subjects; among inapparent cases lymphocyte counts were normal and only 20% were positive for NS1 antigen. Primary dengue infection and a specific dengue virus serotype were not associated with symptomatic dengue infection.

**Conclusion:**

Household investigation demonstrated a high proportion of household members positive for dengue infection, including a number of inapparent cases, the frequency of which was higher in SEA than in LA.

## Author Summary

Dengue is the most important mosquito-borne viral disease in humans. This disease is now endemic in more than 100 countries and threatens more than 2.5 billion people living in tropical countries. It currently affects about 50 to 100 million people each year. It causes a wide range of symptoms, from an inapparent to mild dengue fever, to severe forms, including dengue hemorrhagic fever. Currently no specific vaccine or antiviral drugs are available. We carried out a prospective clinical study in South-East Asia and Latin America, of virologically confirmed dengue-infected patients attending the hospital, and members of their households. Among 215 febrile dengue subjects, 177 agreed to household investigation. Based on our data, we estimated the proportion of dengue-infected household members to be about 45%. At the time of the home visit, almost three quarters of (29/39) presented an inapparent dengue infection. The proportion of inapparent dengue infection was higher in South-East Asia than in Latin America. These findings confirm the complexity of dengue disease in humans and the need to strengthen multidisciplinary research efforts to improve our understanding of virus transmission and host responses to dengue virus in various human populations.

## Introduction

Dengue is the most important mosquito-borne viral disease of humans. The disease is now endemic in more than 100 countries and threatens more than 2.5 billion people. It currently affects about 50 to 100 million people each year [1]. Dengue viruses (DENV) are enveloped, single-stranded positive-sense RNA viruses (family *Flaviviridae*, genus *Flavivirus*). There are four types of DENV: DENV-1, DENV-2, DENV-3 and DENV-4. Dengue virus infection induces life-long protective immunity to the homologous serotype, but confers only partial and transient protection against subsequent infections with any of the other three serotypes [2]. The disease spectrum ranges from inapparent infection or mild dengue fever [3], probably the most common form, to a potentially severe form of dengue characterized by plasma leakage and hemorrhage, known as severe dengue. Uncommonly, severe dengue may manifest as hepatitis, encephalopathy or rhabdomyolysis [2], [4]–[7]. About 500,000 people are estimated to have severe dengue and about 25,000, mostly children, die from it each year [8]. The underlying causes determining the outcome of DENV infection remain unknown. Although previous exposure, viral strain and human host genetic polymorphisms also influence the clinical outcome of DENV infection, we still know little about the complex interplay between host and pathogen in the pathogenesis of dengue [9]–[12].

Inapparent infections have largely been detected retrospectively through serology. The uses of genome detection or virus isolation have enabled detection of inapparent infections in cluster studies designed to detect natural infections in the community [13], [14]. The present study was designed to identify symptomatic and inapparent dengue-infected subjects in genetically-related individuals living in the same household, in line with the main aim of the DENFRAME project which is to explore the influence of human genetic variants and their functional roles in the pathogenesis of dengue disease in humans. We based the identification of dengue-infected subjects upon virological techniques, namely virus isolation and detection of the genome. We also took this opportunity to evaluate prospectively a commercial NS1 capture assay [15], [16] that could potentially be implemented in laboratories for the diagnosis of acute dengue [17]–[19].

## Methods

### Objectives

A multinational, prospective study was conducted in South-East Asia (Cambodia and Vietnam) and Latin America (Brazil and French Guiana). We used virological techniques to identify dengue patients diagnosed at the acute phase of disease among the patients presenting with dengue-like illness. We then performed a household investigation, comparing clinical data and biological markers from subjects with a broad range of dengue disease patterns, including inapparent dengue cases that are rarely captured in clinical studies. This clinical study's aims were: (i) to estimate the proportion of inapparent dengue infections among members of the households of laboratory-confirmed symptomatic dengue cases, (ii) to calculate the proportion of dengue-infected subjects at the time of the household investigation, and (iii) to compare clinical and biological data from inapparent and symptomatic dengue-infected subjects.

### Study sites

Five institutions were involved in this study during the recruitment period: Instituto Evandro Chagas (IEC) in Belém (Pará state, Brazil), *Institut Pasteur du Cambodge* (IPC) in Phnom Penh (Cambodia), *Institut Pasteur de la Guyane* (IPG) in Cayenne (French Guiana) and *Institut Pasteur de Ho Chi Minh Ville* (IPHCM) in Vietnam were responsible for the recruitment of patients and virological analyses; the *Institut Pasteur* (IP) in Paris (France) designed the study and was responsible for central monitoring and data analysis.

**Figure 1:**
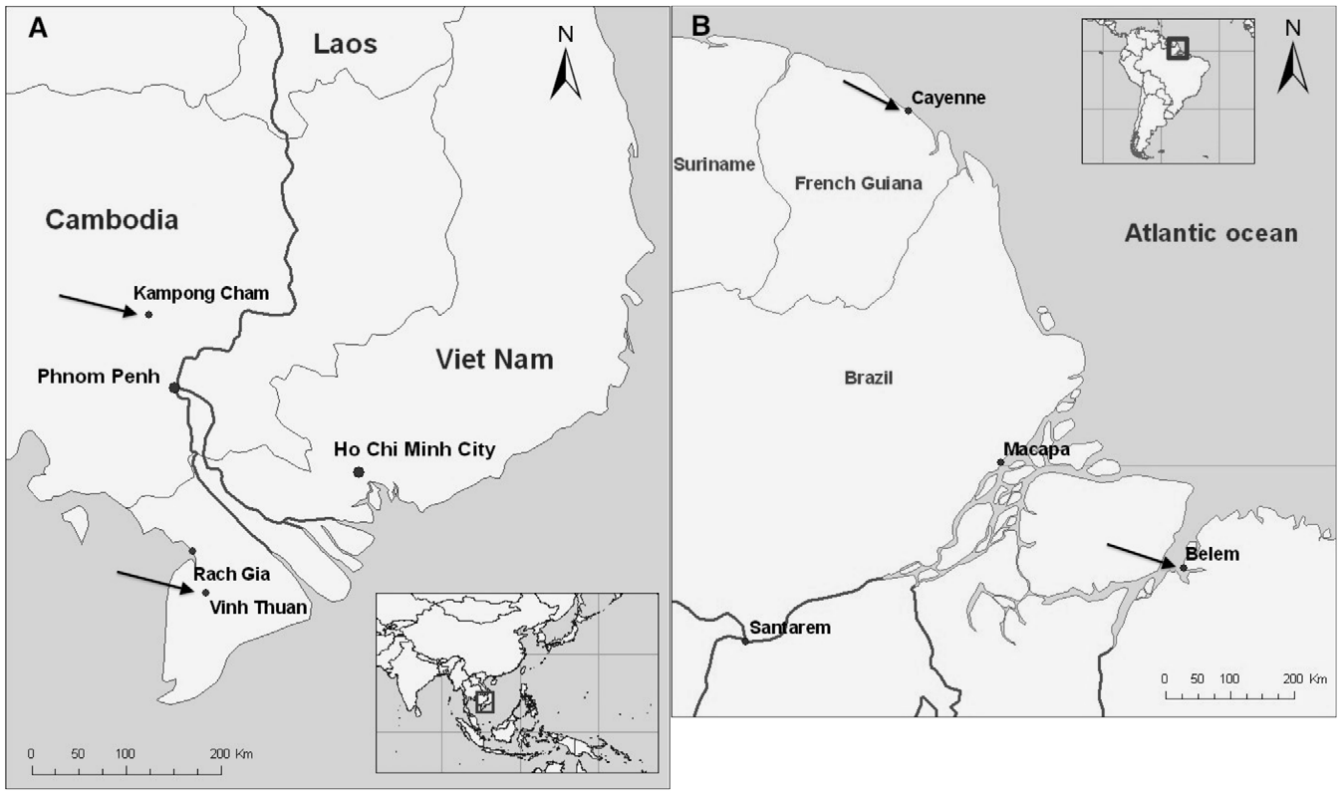
Localization of the four clinical sites. A: in South-East Asia (Cambodia and Vietnam). B in Latin America (Brazil and French Guiana).

As shown in the two maps (Figure 1), volunteers were recruited at four clinical sites: Vinh Thuan District Hospital (Vietnam), Kampong Cham Referral Hospital (Cambodia), the IPG in Cayenne (French Guiana) and public outpatient and emergency rooms managed by the Belém Health Secretariat in the districts of Guamá, Marco, Marambaia and Sacramenta, and the outpatient unit of the IEC (Brazil). The virology laboratories of the four institutions responsible for recruitment are all National Reference Centers (NRC) for Arboviruses (IEC is also a WHO collaborative center). These laboratories carried out virological, NS1 antigen (Platelia Dengue NS1 Antigen, Bio-Rad, Marnes La Coquette, France), and serological techniques.

### Study design

We recruited subjects with acute dengue-like illness at the study sites. These subjects were identified by the treating physicians and were included if they satisfied the following criteria: (i) aged over 24 months; (ii) oral temperature >38°C and onset of symptoms within the last 72 h; and (iii) presenting with at least one clinical manifestation suggestive of dengue-like illness: severe headache, retro-orbital pain, myalgia, joint pain, rash or any bleeding symptom. Furthermore, for inclusion in the second step of the study, the subject had to come from a familial household containing more than two people during the seven days preceding illness. We first identified the dengue-infected subjects (referred to in this study as Dengue Index Cases or DIC) and non-dengue-infected subjects (defined as Non-Dengue Cases - NDC) on the basis of virological results from an acute sample (see below). We then recruited individuals from the households of the DIC. We thus constituted three groups of participants: 1) DIC, 2) household members (HHM), and 3) NDC not related to the DIC. For all groups (DIC, HHM and NDC), we applied the same exclusion criteria: women who were pregnant or breastfeeding, individuals with a focal source of infection (e.g. otitis media, pneumonia, meningitis), patients presenting with a known chronic illness, and patients with malaria. Moreover, to ensure the feasibility of this study, each study site was asked to target a convenient sample of 50 households and to recruit subjects from July 2006 to June 2007 in line with the approval granted by the Institutional Review Board and the timing of the dengue season at each site.

### Clinical data and blood sample collection

**Figure 2:**
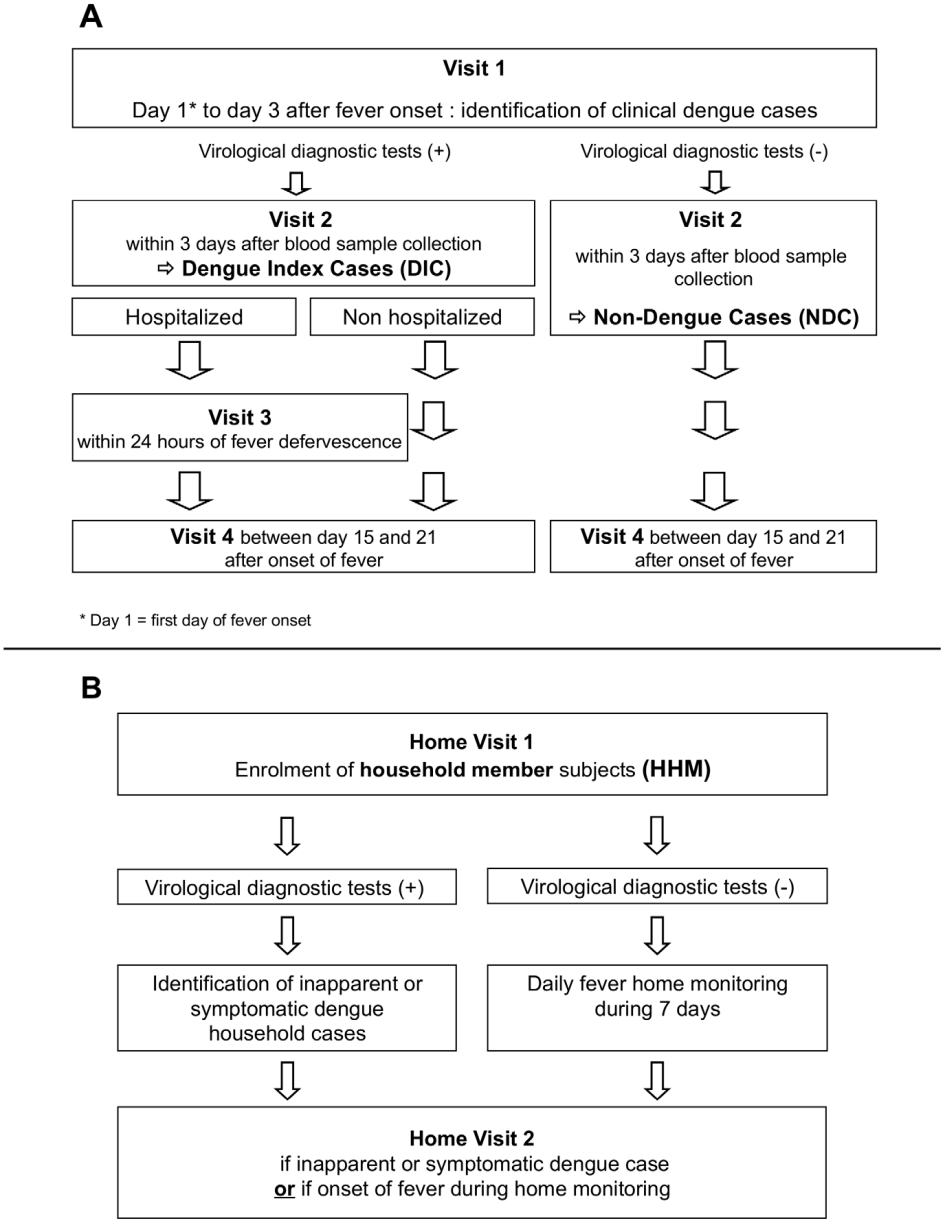
Study design for the inclusion of patients. A: Step 1, identification of dengue index cases (DIC) and non-dengue-infected cases (NDC). B: Step 2, Identification of household members (HHM).

Participants were examined during sequential visits, as shown in the study design charts (Figure 2). At each visit, data were collected with a standardized questionnaire. Severe dengue cases were classified, according to WHO recommendations on the basis of the clinical data. Biological data were also recorded at the sequential visits [2]. Blood samples were collected during the visits and were rapidly processed by the laboratories of each of the recruiting sites, for dengue diagnosis and biological testing. Blood sample volume was adapted for children weighing less than 20 kg.

Paired blood samples were collected for subjects presenting dengue-like illness to allow classification as DIC or NDC: during the acute phase (Visit 1) and during the convalescence phase (Visit 4: 15 to 21 days after the onset of fever). Blood samples were taken from hospitalized DIC within 24 hours of defervescence (Visit 3). HHM were visited at home for blood collection within 24 to 72 hours of DIC identification (Home Visit 1). For practical and logistical reasons this delay of up to 72 hours was unavoidable. HHM were supplied with a monitoring diary card and a thermometer, to enable them to follow their temperature over a 7-day period. For HHM with a positive diagnosis of dengue or with an onset of fever during the seven days of monitoring, a second visit with blood collection for dengue diagnosis was organized (Home Visit 2). Blood analyses included virological and serological dengue diagnosis, complete blood count, transaminases and bilirubin levels. Finally, the data were coded and entered into the computer via a secure website specifically developed with the PHP/MySQL system.

### Classification of dengue cases on the basis of acute dengue diagnosis

All serum samples collected at Visit 1 or at Home Visit 1 or Home Visit 2 were tested: (i) for acute dengue diagnosis, defined as positive virus isolation on mosquito cells [20] and/or positive viral RNA detection by reverse transcriptase-polymerase chain reaction (RT-PCR) [21], and (ii) for the diagnosis of early convalescent dengue cases based on a standardized DENV IgM capture enzyme-linked immunosorbent assay (MAC-ELISA) [22], and DENV IgG detection by indirect ELISA (in-house protocol developed by each NRC for Arboviruses). NS1 antigen detection was also performed.

Only subjects with febrile dengue infection diagnosis were classified as DIC. Subjects in the early stage of dengue convalescence at Visit 1 (*i.e.* positive NS1 antigen detection with concomitant DENV IgM detection, or isolated DENV IgM detection with no positive viral tests) were not classified as DIC; we did not perform a household investigation for them. For the classification of dengue-infected HHM at Home Visit 1, we included both HHM with an acute (febrile or inapparent) dengue infection diagnosis and HHM with isolated DENV IgM detection, presumably related to an infection preceding that of the DIC (i.e in the early convalescence phase). During the 7-day period of home monitoring, several new febrile cases of dengue-infected HHM were also confirmed through Home Visit 2.

We were unable to use the DENV IgM/IgG ratio to distinguish between primary and secondary dengue infections, due to a lack of standardization of DENV IgG tests among laboratories [23]. We therefore established two groups of dengue-infected participants, based on the presence or absence of DENV IgG during the acute phase of the disease. In this study, we considered the presence of DENV IgG in the acute phase of the study to be suggestive of previous dengue infection. All sera were also checked for DENV IgM and IgG at Visit 4. Finally, if all these dengue tests were negative, participants were classified as NDC.

### Ethics

The study was approved by the Institutional Review Board of the *Institut Pasteur* and by the ethics committees of each of the countries concerned. It was conducted in accordance with the Declaration of Helsinki, and the participants or the parents of minors participating in the study gave written informed consent before inclusion. The clinical protocol, the questionnaires, the standard operating procedures and informed consent forms were adapted and translated for each clinical site. All the documentation was accessible through a dedicated website with a specific login access (www.denframe.org). The centralized electronic database was based at the *Institut Pasteur* in Paris and registered with the *Commission Nationale de l'Informatique et des Libertés* (CNIL) in France.

### Statistical methods

We present here the data from all four study sites in Latin America and South-East Asia. DIC are described according to region, disease severity, DENV type, age group and IgG status. We estimated the proportion of inapparent dengue infections among HHM, and we calculated the proportions of dengue-infected subjects among household subjects, in total and according to the IgG status at the time of household investigation. We compared clinical data and biological markers between inapparent dengue-infected subjects, symptomatic dengue-infected subjects, and non-dengue-infected participants at the time of the household investigation. We created binary variables to evaluate the potential effect of DENV infection on biological markers (hematocrit, platelets, neutrophils, lymphocytes, monocytes, ASAT, ALAT, bilirubin). For lymphocytes and neutrophils, we used a threshold of 2×10^9^/l. We used chi-squared or Fisher's exact tests to compare categorical variables between symptomatic cases, inapparent dengue-infected cases and non-dengue-infected subjects among HHM. Univariate and multivariable logistic regression models were used to assess the effect of covariates on the odds ratios (OR) of symptomatic dengue-infected cases, inapparent dengue-infected cases, and non-dengue-infected subjects among HHM. For the multivariable logistic regression models including data from household members, we used two-stage hierarchical regression models taking into account the family household structure [24]. Potential confounders with a P value of less than 0.20 in univariate analysis were retained for the final multivariable analyses. STATA version 10.0 (Stata Corp., College Station, TX, USA) and a significance level of 5% were used for all statistical analyses.

## Results

**Figure 3:**
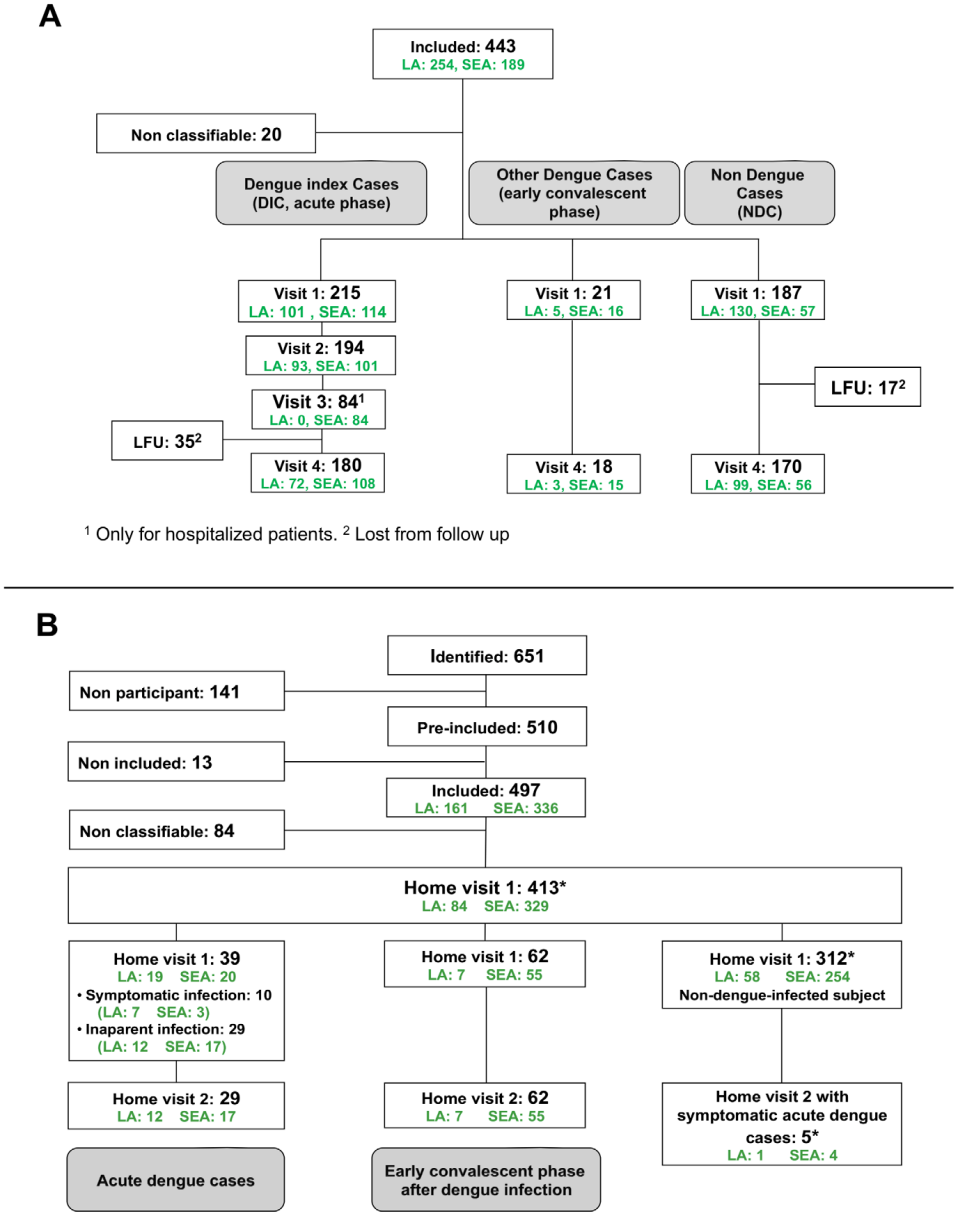
Identification of the dengue index cases (DIC) and of the household members (HHM). A: Identification of DIC in Step 1. B: Recruitment of HHM for 177 DIC during Step 2. * Full assessment of DENV infection was performed for a total of 413 HHM at Home Visit 1, and 312 subjects were considered as non-dengue-infected at that time. Five of them developed a dengue fever and were excluded from our analysis, defining a total of 408 HHM at Home Visit 1. Among them, 307 (312 - 5) subjects may have had an inapparent dengue infection after Home Visit 1 as we did not perform blood sample collection at Home Visit 2 for non-symptomatic subjects.

Flowcharts for the recruitment of participants at each step are shown in Figure 3.

### Step 1: identification of dengue index cases (DIC)

We screened 473 febrile subjects for dengue infection. Thirty (6.3%) had at least one criterion for non inclusion in the study at presentation; the remaining 443 (93.7%) were included in the study. We identified 215 (48.5%) of these 443 subjects as DIC, 21 (4.7%) as dengue convalescent cases, 187 (42.2%) as NDC, and 20 (4.5%) could not be classified because some biological markers were lacking. Recruitment levels during the study period were very low in French Guiana (9 DIC and 24 NDC), whereas there had been a large number of dengue cases during the rainy season of the previous year [25]. For the 215 subjects classified as DIC, 149 (69.3%) were positive by genome detection and viral isolation, 43 (20.0%) were positive by genome detection only, 15 (7.0%) were positive by viral isolation only, and a very few subjects (n = 8, 3.7%) were ultimately classified as DIC by the virologists, based on positive NS1 detection, clinical data and serological results (negative IgM at Visit 1 followed by seroconversion IgM at convalescent phase).

The proportions of subjects classified as either NDC or DIC differed between Latin America and South-East Asia: 69.5% (130/187) of the total NDC in the study, and 47.0% (101/215) of the DIC, were recruited in Latin America whereas 30.5% (57/187) of the NDC and 53.0% (114/215) of the DIC were recruited in South-East Asia (P<10^−4^) (Figure 3A). In other words, in Latin America, in two thirds of subjects presenting with dengue-like illness, the cause was not related to dengue infection. Given the inclusion criteria, the dengue-like illness symptoms were not different between NDC and DIC (data not shown). However, all biological variables, including counts of platelets, lymphocytes and neutrophils, were significantly lower, whereas hematocrit and liver enzyme levels were higher in the DIC group than in the NDC group (data not shown).

Table 1 shows the distribution of DIC by region and according to IgG status at Visit 1 as a function of DENV type and age group. The proportions of severe dengue and dengue fever cases with DENV IgG (suggestive of previous DENV infection) and without DENV IgG in the acute phase were similar (Table 1): 15 (55.6%) severe dengue cases tested negative for DENV IgG and 12 (44.4%) tested positive for DENV IgG, versus 49 (31.8%) and 105 (68.2%) of the subjects with non severe disease, respectively (P = 0.017). DENV-1, -2 and -3 were found with similar frequencies in South-East Asia, whereas DENV-3 predominated in Latin America. Fifteen of the severe dengue cases reported in South-East Asia were infected with DENV-2 (53.6%; 15/28). Interestingly, seven severe dengue cases positive for DENV-2 virus and negative for DENV IgG in the acute phase but with subsequent DENV IgM and IgG seroconversion were identified. This serological pattern suggests that these patients had primary DENV infection. Two DIC in Vietnam were reported with co-detection of multiple DENV strains by RT-PCR: DENV-2/DENV-1 and DENV-4/DENV-2 respectively; the viral cultures were negative for both subjects. Only the first virus detected was considered for further statistical analysis (DENV-2 and DENV-4, respectively).

**Table 1:** Characteristics of dengue index cases (DIC, n = 215).

	Acute serum samples (n = 215)											
	Latin America (n = 101)						South-East Asia (n = 114)*					
	Negative IgG (n = 14)			Positive IgG (n = 87)			Negative IgG (n = 60)			Positive IgG (n = 51)		
	Severe denguen = 0 (%)	Dengue fevern = 6 (%)	Non classifiablen = 8 (%)	Severe denguen = 0 (%)	Dengue fevern = 70 (%)	Non classifiablen = 17 (%)	Severe denguen = 15 (%)	Dengue fevern = 43 (%)	Non classifiablen = 2 (%)	Severe denguen = 12 (%)	Dengue fevern = 35 (%)	Non classifiablen = 4 (%)
**Dengue type**												
DENV-1	-	3 (50.0)	-	-	8 (11.4)	3 (17.6)	4 (26.7)	20 (46.5)	-	2 (16.7)	14 (40.0)	-
DENV-2	-	-	8 (100.0)	-	13 (18.7)	1 (5.9)	7 (46.7)	12 (27.9)	-	7 (58.3)	6 (17.2)	-
DENV-3	-	3 (50.0)	-	-	47 (67.1)	13 (76.5)	3 (20.0)	9 (21.0)	1 (50.0)	1 (8.3)	11 (31.4)	2 (50.0)
DENV-4	-	-	-	-	-	-	-	1 (2.3)	-	-	2 (5.7)	1 (25.0)
Missing data	-	-	-	-	2 (2.8)	-	1 (6.6)	1 (2.3)	1 (50.0)	2 (16.7)	2 (5.7)	1 (25.0)
**Age-group (years)**												
[2–7]	-	-	-	-	3 (4.3)	2 (11.8)	2 (13.3)	21 (48.9)	2 (100.0)	2 (16.7)	13 (37.1)	-
[7–10]	-	1 (16.7)	1 (12.5)	-	-	1 (5.9)	3 (20.0)	9 (20.9)	-	4 (33.3)	6 (17.1)	1 (25.0)
>10	-	5 (83.3)	7 (87.5)	-	67 (95.7)	13 (76.4)	10 (66.7)	13 (30.2)	-	6 (50.0)	16 (45.7)	3 (75.0)
Missing data	-	-	-	-	-	1 (5.9)	-	-	-	-	-	-

*For 3 subjects infected by DENV-2, data related to IgG status were missing: 2 dengue fever cases and 1 severe dengue case.

Distribution of DIC is provided by region in relation to the presence of WHO criteria for severe dengue and IgG status during the acute phase.

According to the WHO criteria, twenty-eight (13.0%) subjects were classified as severe dengue (based on severe plasma leakage and/or severe hemorrhages and/or severe organ impairment). All these cases were from clinical sites in South-East Asia (25 in Vietnam and 3 in Cambodia, as presented in Table S1). At visit 1, presentation with the following combination of features was significantly associated with the occurrence of severe dengue in this population: being male, over the age of seven years, with no retro-orbital pain but with bleeding, low monocyte count, normal liver enzyme levels and DENV-2 type infection.

For 163 (75.8%) DIC, data were available for all the biological markers at visits 1 and 4 (Figure 3A). All these markers had returned to normal levels by visit 4, and all participants, including the 28 severe dengue cases displayed clinical recovery from dengue disease (data not shown).

### Step 2: identification of household members (HHM)

Agreement for household investigations was obtained from 177 (82.3%) DIC, corresponding to a total of 651 household members. We compared the distribution of the covariates (as listed in Table S1) between the 38 DIC with no familial investigation and the 177 DIC who underwent familial investigation; no significant differences were found in the distribution of the covariates between these two groups (data not shown). All 28 patients with severe dengue infection underwent household investigation. In total, 141 (21.7%) of the 651 household members refused to participate in the study. We therefore screened 510 participants, 497 (97.5%) of whom were eligible for the study. All but one of these 497 household members were genetically related to the DIC. Eighty-four were not classifiable due to the lack of some biological results. Full assessment of DENV infection was carried out according to the study protocol for the remaining 413 of these subjects (Figure 3B) during Home Visit 1.

At the time of the household investigation (Home Visit 1), 39 subjects were identified as being in the acute phase of dengue infection: 29 (74.4%) cases were inapparent and 10 (25.6%) had symptomatic dengue infection. An additional 62 subjects were classified as being in the early phase of convalescence from dengue infection. The remaining 312 subjects were considered as non-dengue-infected at the time of Home Visit 1 (Figure 3B); however, five of them developed some clinical symptoms of dengue fever and were laboratory-confirmed as having acute dengue infection during the 7-day home monitoring. We excluded them (n = 5) from the remaining analysis (n = 312 subjects with 7-day home monitoring) that thus included 307 subjects (Figure 3B). It should be noted that a second home visit and blood sampling was not possible, for ethical and logistical reasons, for HHM without any clinical symptoms after the 7-day home monitoring. Hence, among the 307 remaining subjects, some may have had an inapparent dengue infection after Home Visit 1. Therefore, we considered that at least 101 (39 acute or 62 early convalescent) dengue infections were found amongst 408 HHM (24.8%; 95% confidence interval (CI): 20.6–28.9) at the time of Home Visit 1 (Figure 3B). Thus, adding together the 177 DIC and the 101 DENV-infected HHM, the overall proportion for dengue among the study participants was estimated at 47.5% (278/585; 95% CI: 43.5–51.6) (Figure 3B). We have also estimated these proportions according to the IgG status (Table 2) at the time of Home Visit 1 (excluding the 5 subjects with known symptomatic infection – 3 were IgG positive and 2 were IgG negative). Among the 585 subjects, 6 had missing IgG data. Among 425 subjects with positive IgG, the estimated proportion of dengue-infected subjects was 43.8% (186/425; 95% CI: 39.0–48.5) and, among the 154 with negative IgG, this estimated proportion was 57.1% (88/154; 95% CI: 49.3–65.0).

**Table 2:** Distribution of the participants in the clinical study (n = 590).

	Brazil	French Guiana	Cambodia	Vietnam	Total
	n = 134 (%)	n = 28 (%)	n = 180 (%)	n = 248 (%)	n = 590 (%)
	[IgG+/IgG−]	[IgG+/IgG−]	[IgG+/IgG−]	[IgG+/IgG−]	[IgG+/IgG−]
Non DENV-infected subjects	47 (15.4)	9 (3.0)	98 (32.1)	151 (49.5)	**305 (51.7)**
	[44/3]	[3/6]	[95/3]	[97/54]	**[239/66]**
Missing IgG data	1	-	-	1	**2 (0.3)**
Early convalescent phase or convalescent phase (HHM only)	4 (6.5)	3 (4.9)	22 (36.1)	32 (52.5)	**61 (10.3)**
	[4/0]	[2/1]	[22/0]	[25/7]	**[53/8]**
Missing IgG data	-	-	-	1	**1 (0.2)**
DENV-infected at the acute phase (DIC+HHM)	82 (37.6)	16 (7.4)	60 (27.5)	60 (27.5)	**218 (37.0)**
Symptomatic	[69/6]	[3/10]	[30/19]	[16/36]	**[118/71]**
Missing IgG data	-	-	-	3	**3 (0.5)**
Inapparent dengue infection	[6/1]	[1/2]	[8/3]	[3/5]	**[18/11]**

All participants were identified at Visit 1 for Dengue Index Cases (DIC) and at Home Visit 1 for dengue-infected household members (HHM). Their distribution is presented by country, according to DENV-infected status and IgG status.

In 101 (57.1%) households, there was only one dengue-infected case. For the 76 (42.9%) households with at least two dengue-infected cases, DENV type had been determined for all subjects in 29 households. Nine (31.0%) households were found to have two different DENV types circulating during the same time period: DENV-1 & DENV-3 (n = 2 in Brazil, n = 4 in Cambodia), DENV-1 & DENV-2 (n = 1 in Vietnam), and DENV-2 & DENV-3 (n = 2 in Vietnam).

Hematologic and hepatic biological markers observed among non-dengue-infected cases (n = 307), inapparent dengue-infected cases (n = 29), and symptomatic dengue-infected subjects (n = 192) are described in Table S2. Tables 3 & 4 show comparisons between non-dengue-infected and inapparent dengue-infected cases, and symptomatic and inapparent dengue-infected subjects, respectively, among the household subjects. Table S3 presents the main characteristics of subjects with acute dengue infection compared to non-dengue-infected subjects among the household subjects. In the comparisons between non-dengue-infected and inapparent dengue-infected subjects, taking into account potential confounders, only neutrophil and monocyte levels differed significantly whereas presence of IgG at Visit 1 was almost significant with the non-dengue-infected group. The comparison between symptomatic and inapparent dengue-infected subjects (Table 4) showed significant difference between groups for lymphocyte counts and positive NS1 antigen detection. In this analysis, no significant difference was found for DENV types identified or IgG detection during the acute phase.

**Table 3:** Main characteristics of subjects with inapparent dengue infections compared to non-dengue-infected subjects among Household members.

	Non-dengue-infectedn = 307 (%)	Inapparent dengue infectionn = 29 (%)	Crude OR	95% CI	P*	Adjusted OR	95% CI	P
**Sex**								
Male	135 (44.0)	16 (55.2)	1					
Female	172 (56.0)	13 (44.8)	0.64	[0.3–1.4]	0.25			
**Age (years)**								
[2–7]	16 (5.2)	5 (17.2)	1			1		
[7–10]	17 (5.5)	2 (6.9)	0.38	[0.1–2.2]	0.28	0.79	[0.1–6.5]	0.83
>10	274 (89.3)	22 (75.9)	0.26	[0.1–0.7]	0.015	0.41	[0.1–1.8]	0.25
**Weight-based Z-score**								
[−1, 1]	89 (29.0)	6 (20.7)	1					
<−1	195 (63.5)	21 (72.4)	1.6	[0.6–4.1]	0.33			
>1	23 (7.5)	2 (6.9)	1.3	[0.2–6.8]	0.76			
**Hematocrit (%)**								
≤36	93 (30.3)	7 (24.1)	1					
>36	212 (69.1)	22 (75.9)	1.38	[0.6–3.3]	0.48			
Missing data	2 (0.6)	-						
**Platelets (×10^9^/L)**								
>100	296 (96.4)	26 (89.7)	1			1		
≤100	10 (3.3)	3 (10.3)	3.42	[0.9–13.2]	0.075	1.71	[0.2–12.3]	0.6
Missing data	1 (0.3)	-						
**Neutrophils (×10^9^/L)**								
>2	288 (93.8)	18 (62.1)	1			1		
≤2	18 (5.9)	11 (37.9)	9.8	[4–23.8]	<0.0001	7.75	[2.5–24]	**<0.0001**
Missing data	1 (0.3)	-						
**Lymphocytes (×10^9^/L)**								
>2	243 (79.2)	15 (51.7)	1			1		
≤2	63 (20.5)	14 (48.3)	3.6	[1.6–7.8]	0.001	2.08	[0.7–5.6]	0.15
Missing data	1 (0.3)	-						
**Monocytes (×10^9^/L)**								
>0.2	298 (97.1)	23 (79.3)	1			1		
≤0.2	8 (2.6)	6 (20.7)	9.72	[3.1–30]	<0.0001	9.1	[1.8–44]	**0.006**
Missing data	1 (0.3)	-						
**ASAT** a **(UI/L)**								
≤30	225 (73.3)	17 (58.6)	1			1		
>30	81 (26.4)	11 (37.9)	1.8	[0.8–4]	0.15	1.96	[0.7–5.2]	0.17
Missing data	1 (0.3)	1 (3.5)						
**ALAT** b **(UI/L)**								
≤35	261 (85.0)	22 (75.9)	1					
>35	45 (14.7)	6 (20.7)	1.58	[0.6–4.1]	0.35			
Missing data	1 (0.3)	1 (3.4)						
**Bilirubin (µmol/L)**								
≤17	262 (85.3)	24 (82.8)	1					
>17	42 (13.7)	3 (10.3)	0.78	[0.2–2.7]	0.69			
Missing data	3 (1.0)	2 (6.9)						
**IgG at Visit 1**								
Negative	66 (21.5)	11 (37.9)	1			1		
Positive	239 (77.8)	18 (62.1)	0.45	[0.2–1.0]	0.051	0.37	[0.1–1.04]	0.06
Missing data	2 (0.7)	-						

*Potential confounders with a P value of less than 0.20 in univariate analysis were retained for the final multivariable analyses. In this table: age, platelets, neutrophils, lymphocytes, ASAT and IgG at Visit 1.

a ASAT: Aspartate amino transferase.

b ALAT: Alanine amino transferase.

Univariate and multivariable logistic regression were used for analyses.

**Table 4:** Main characteristics of subjects with inapparent dengue infections compared to symptomatic dengue-infected subjects.

	Symptomatic dengue-infectedn = 192 (%)	Inapparent dengue infectionn = 29 (%)	Crude OR	95% CI	P*	Adjusted OR	95% CI	P
**Sex**								
Male	103 (53.6)	16 (55.2)	1					
Female	89 (46.4)	13 (44.8)	0.94	[0.4–2.1]	0.88			
**Age (years)**								
[2–7]	38 (19.8)	5 (17.2)	1					
[7–10]	27 (14.1)	2 (6.9)	0.56	[0.1–3.1]	0.51			
>10	127 (66.1)	22 (75.9)	1.32	[0.5–3.7]	0.6			
**Weight-based Z-score**								
[−1, 1]	75 (39.1)	6 (20.7)	1			1		
<−1	102 (53.1)	21 (72.4)	2.57	[0.9–6.7]	0.052	2.54	[0.6–10.4]	0.20
>1	15 (7.8)	2 (6.9)	1.66	[0.3–9.1]	0.55	4.11	[0.4–43]	0.24
**Hematocrit (%)**								
≤36	38 (19.8)	7 (24.1)	1					
>36	154 (80.2)	22 (75.9)	0.77	[0.3–1.9]	0.59			
**Platelets (×10^9^/L)**								
>100	126 (65.6)	26 (89.7)	1			1		
≤100	66 (34.4)	3 (10.3)	0.22	[0.1–0.7]	0.016	0.23	[0.4–1.4]	0.11
**Neutrophils (×10^9^/L)**								
>2	76 (39.6)	18 (62.1)	1			1		
≤2	116 (60.4)	11 (37.9)	0.4	[0.2–0.9]	0.026	0.5	[0.15–1.6]	0.25
**Lymphocytes (×10^9^/L)**								
>2	16 (8.3)	15 (51.7)	1			1		
≤2	176 (91.7)	14 (48.3)	0.08	[0.03–0.2]	<0.0001	0.09	[0.02–0.4]	**0.001**
**Monocytes (×10^9^/L)**								
>0.2	114 (59.4)	23 (79.3)	1			1		
≤0.2	78 (40.6)	6 (20.7)	0.38	[0.1–0.9]	0.045	0.65	[0.16–2.7]	0.56
**ASAT** a **(UI/L)**								
≤30	75 (39.1)	17 (58.6)	1			1		
>30	117 (60.9)	11 (37.9)	0.4	[0.2–0.9]	0.034	0.4	[0.1–1.5]	0.17
Missing data	-	1 (3.5)						
**ALAT** b **(UI/L)**								
≤35	112 (58.3)	22 (75.9)	1			1		
>35	80 (41.7)	6 (20.7)	0.38	[0.15–0.9]	0.046	0.52	[0.14–1.9]	0.33
Missing data	-	1 (3.4)						
**Bilirubin (µmol/L)**								
≤17	175 (91.1)	24 (82.8)	1					
>17	14 (7.3)	3 (10.3)	1.56	[0.4–5.8]	0.51			
Missing data	3 (1.6)	2 (6.9)						
**DENV type**								
DENV-1	50 (26.0)	5 (17.2)	1					
DENV-2	50 (26.0)	7 (24.2)	1.4	[0.4–4.7]	0.59			
DENV-3	79 (41.2)	13 (44.8)	1.64	[0.5–4.9]	0.37			
DENV-4	3 (1.6)	-						
Missing data	10 (5.2)	4 (13.8)						
**IgG at Visit 1**								
Negative	71 (37.0)	11 (37.9)	1					
Positive	118 (61.4)	18 (62.1)	0.98	[0.4–2.2]	0.97			
Missing data	3 (1.6)	-						
**NS1 antigen**								
Negative	21 (10.9)	23 (79.3)	1			1		
Positive	171 (89.1)	6 (20.7)	0.03	[0.01–0.1]	<0.0001	0.05	[0.01–0.2]	**<0.0001**

*Potential confounders with a P value of less than 0.20 in univariate analysis were retained for the final multivariable analyses. In this table: weight-based Z-score, platelets, neutrophils, lymphocytes, monocytes, ASAT, ALAT and NS1 antigen.

a ASAT: Aspartate amino transferase.

b ALAT: Alanine amino transferase.

Univariate and multivariable logistic regression were used for analyses.

## Discussion

Several previous epidemiological studies have focused on school-based surveillance aiming at improving dengue-vector control measures [3], [14], studying the dynamics of patterns of dengue transmission [26]–[28] or describing a model that takes into account the role of human movement in the transmission dynamics of vector-borne pathogens [29]. Earlier cluster investigation methods were designed as an alternative approach to the commonly used prospective cohort study method for investigating the natural history of dengue virus infection in South-East Asia and Latin America [13], [30]. Although different study designs have demonstrated the feasibility of identification of inapparent dengue cases, it remains difficult to recruit these subjects. We designed our study to include family household investigation in order to identify a group of inapparent dengue-infected subjects and to compare them with symptomatic dengue-infected and non-dengue-infected subjects living in the same family household. The study design was based on family household recruitment specifically in order to collect data and biological samples, and to study secondarily the host susceptibility to dengue infection and disease. Unlike studies based on cohorts from hospital referrals, this multi-country study captured dengue cases ranging from inapparent infections, through mild disease to severe dengue fever, using definitions of clinical cases and diagnostic methodology standardized across the four sites. The period of inclusion, from July 2006 to June 2007, spanned the dengue season at each site, although incidence of dengue was low that year in French Guiana.

The main objective of this study was to identify dengue infections and particularly inapparent infections among dengue patients' household family members in South-East Asia and Latin America. Based on our data, we estimated the proportion to be about 45% among those participating in the household study. Most of the dengue cases studied had symptomatic infections, covering the spectrum of disease from dengue fever to severe dengue cases. We also identified inapparent infections in the population. We observed dengue-infected subjects classified as DIC and some of their HHM without acute dengue infection but with a positive IgM detection, suggesting an early convalescent phase after dengue infection with no clinical symptoms. In this study we identified 29 inapparent dengue infections but we believe this number underestimates the proportion of inapparent dengue cases because we were not able to take blood samples from non-symptomatic subjects at Home Visit 2.

We postulated that dengue is transmitted to members of the DIC's family household during the period of the index subject's infection, and thus designed our study to detect inapparent dengue infections with a home visit organized shortly after identification of DIC. Obviously, we cannot confirm whether the index subject's DIC was always the source of infection in other family members, but we can postulate that a non-hospitalized DIC who remains at home during acute illness represents a potential source of DENV transmission to *Aedes*. According to our study design, clustering of cases within a household could be the result of a single or very few infected mosquitoes biting different household members during a short period of time, perhaps within a single gonotrophic cycle as previously suggested [14], [31]. This is also consistent with a previous observation that over periods from 1 to 3 days, dengue cases were clustered within short distances, i.e., within a household [32]. No mosquito captures were, however, conducted in our study to identify DENV-positive *Aedes* mosquitoes. DENV sequencing would help resolve the extent of localized transmission.

We characterized subjects with acute dengue infection using virus isolation and detection of the genome. We also used NS1 antigen detection, a more recently recognised diagnostic tool. As for many tropical infectious diseases, there is an urgent need for validated diagnostic tools for dengue. In parallel with the virological techniques, we evaluated detection of the NS1 antigen with the Platelia Dengue NS1 Ag test. In this study, this test was found to have good sensitivity (83.6%; 95% CI: 78.5–88.6) and specificity (98.9%; 95% CI: 96.6–99.9) in both Asia and Latin America, as reported in previous studies [17], [33], [34]. A recent multi-country study observed unequal sensitivity between geographical regions that remains unexplained, suggesting further assessments are needed [35]. The use of viral detection antigen is particularly useful during the first five days of illness with NS1 assays that are significantly more sensitive for primary than secondary dengue [18], [34], [36]. However, NS1 antigen could be detected in only 20% of inapparent DENV-infection. This finding suggests that NS1 antigen may have a role in dengue disease pathogenesis and also indicates that this test cannot be relied upon for detection of inapparent dengue infection.

By comparing HHM not infected with dengue with those presenting with inapparent dengue infection, we showed that neutrophil and monocyte counts were early indirect biological markers of dengue infection, whereas platelet counts and the frequency of IgG detection at the first visit did not differ between the two groups (Table 3). A comparison of inapparent dengue-infected HHM with symptomatic dengue-infected subjects showed that lymphocyte counts and detection of the NS1 antigen differed significantly between these two groups (Table 4). Moreover, the NS1 antigen was detected during the acute phase in most of the dengue cases tested, and the sensitivity of this test was even higher in severe dengue cases (26/28, Table S1), possibly reflecting higher viral loads. These findings may indirectly reflect the progression of the immune response to DENV, leading in some cases to severe acute lymphopenia and a lack of virological control, with high rates of NS1 antigen circulation in the blood that may be correlated with high-level or prolonged viremia [7], [36]. Severe dengue cases were also more likely to be male, to have lower monocyte counts or normal liver enzyme levels, and to be infected with DENV-2, although quantitative RT-PCR did no permit study of the magnitude of the viremia. We showed that half of the severe dengue cases had not previously been infected with DENV, as confirmed by the occurrence of DENV IgG seroconversion during convalescent phase [7]. In all dengue-infected subjects, including inapparent, we observed a decrease in neutrophil and monocyte counts. On one hand, it may suggest a direct effect of dengue illness on hematopoiesis, although such an effect is in conflict with data reported elsewhere in the literature [37]. On the other hand, DENV is detected in peripheral monocytes during acute disease, and the infection of monocytes leads to cytokine production, suggesting that virus-monocyte interactions are relevant to pathogenesis [38]–[40]. Moreover, DENV can induce apoptosis in monocytes, and this may lead to decreases in the number of these cells in severe dengue cases [41].

In this study we only observed severe dengue cases in South-East Asia. Disease severity and pathogenesis remain largely unexplained and certainly related to complex interactions of several factors, including virus strain, immune response to previous dengue infection and host genetic background. The introduction of the Asian 1 DENV-2 genotype into the Americas in the 1980s led to the emergence of severe dengue cases on this continent. Following this introduction a new genotype emerged, named Asian/American DENV-2 genotype [42]–[44]. During the study period, this Asian/American genotype was circulating in French Guiana (Philippe Dussart, personal data) and probably in the north of Brazil, however DENV-2 did not cause an outbreak and we did not report any severe dengue case among Brazilian subjects.

Two constraints of the study design deserve mention. All methods (biological markers, virological testing, NS1 antigen detection and IgM serology) were standardized across the four reference laboratories, with the exception of the IgG ELISA. As a consequence, we were unable to calculate the IgM/IgG ratio [45], [46]. However, as the intention was to include dengue cases during the acute phase of infection, this ratio was not a crucial endpoint for the study. Another constraint of this study was that we did not include infants and children below 24 months of age in the DENFRAME project. However, several previous reports already provide insight into the epidemiology of dengue in this specific population [47]–[50].

These findings confirm the complexity of dengue disease in humans and the need to strengthen multidisciplinary research efforts to improve our understanding not only of virus transmission but also host responses to DENV in various human populations. It will therefore be interesting, based on clinical data and biological samples collected in this study, to further evaluate the host susceptibility to dengue infection and disease using family-based association analyses. Moreover, we think that technological transfer of standardized diagnostic methods in laboratories based in tropical countries is essential if we are to estimate disease burden and to optimize vector control interventions. Together with improvements in clinical care for dengue patients and better understanding of dengue pathogenesis, the development of a preventive vaccine and antiviral drugs would complete the arsenal of weapons for combating dengue worldwide.

## Supporting Information

Checklist S1 STROBE checklist.
(PDF)

Table S1 Characteristics of dengue index cases from South-East Asia based on Visit 1 data (n = 114).
(DOC)

Table S2 Continuous biological markers observed among non-dengue-infected, inapparent dengue infection and symptomatic dengue-infected subjects.
(DOC)

Table S3 Main characteristics of subjects with acute dengue infection compared to non-dengue-infected subjects.
(DOC)
