# Supplementary material for: Clinical and Virological Study of Dengue Cases and the Members of Their Households: The Multinational DENFRAME Project
Source: PLoS Negl Trop Dis. 2012 Jan 24;6(1):e1482. doi: 10.1371/journal.pntd.0001482 (PMC3265457; doi:10.1371/journal.pntd.0001482)
Supplement: Table S1 — Characteristics of dengue index cases from South-East Asia based on Visit 1 data (n = 114). (DOC) [file pntd.0001482.s002.doc]

**Table S1. Characteristics of dengue index cases from South-East Asia based on Visit 1 data (n =114).** This table presents results of univariate analysis adjusted for sites.

|  | Dengue fever | Severe dengue |  |  |  |  |
| --- | --- | --- | --- | --- | --- | --- |
|  | n = 86 (%) | n = 28 (%) |  | OR | 95% CI | P |
| **Sex** |  |  |  |  |  |  |
| Male | 40 (46.5) | 21 (75.0) |  | 1 |  |  |
| Female | 46 (53.5) | 7 (25.0) |  | 0.29 | [0.1-0.7] | **0.01** |
| **Age (years)** |  |  |  |  |  |  |
| [2 - 7] | 37 (43.0) | 4 (14.3) |  | 1 |  |  |
| ]7 - 10] | 16 (18.6) | 7 (25.0) |  | 4.05 | [1.04-16] | **0.04** |
| > 10 | 33 (38.4) | 17 (60.7) |  | 4.76 | [1.4-15.6] | **0.01** |
| **Weight-based Z-score** |  |  |  |  |  |  |
| [-1, 1] | 8 (9.3) | 6 (21.4) |  | 1 |  |  |
| < -1 | 78 (90.7) | 22 (78.7) |  | 0.37 | [0.1-1.2] | 0.09 |
| > 1 | - | - |  | - | - | - |
| **Fever (°C)** |  |  |  |  |  |  |
| ≤ 39 | 69 (80.2) | 26 (92.9) |  | 1 |  |  |
| > 39 | 17 (19.8) | 2 (7.1) |  | 0.31 | [0.07-1.4] | 0.14 |
| **Headache** |  |  |  |  |  |  |
| No | 23 (26.7) | 10 (35.7) |  | 1 |  |  |
| Yes | 63 (73.3) | 18 (64.3) |  | 0.66 | [0.3-1.6] | 0.36 |
| **Retro-orbital pain** |  |  |  |  |  |  |
| No | 51 (59.3) | 24 (85.7) |  | 1 |  |  |
| Yes | 35 (40.7) | 4 (14.3) |  | 0.24 | [0.07-0.7] | **0.015** |
| **Myalgia** |  |  |  |  |  |  |
| No | 66 (76.7) | 22 (78.6) |  | 1 |  |  |
| Yes | 20 (23.3) | 6 (21.4) |  | 0.9 | [0.3-2.5] | 0.84 |
| **Joint pain** |  |  |  |  |  |  |
| No | 79 (91.9) | 24 (85.7) |  | 1 |  |  |
| Yes | 7 (8.1) | 4 (14.3) |  | 1.88 | [0.5-6.9] | 0.34 |
| **Bleeding symptom** |  |  |  |  |  |  |
| No | 46 (53.5) | 5 (17.9) |  | 1 |  |  |
| Yes | 40 (46.5) | 23 (82.1) |  | 5.29 | [1.8-15.2] | **0.002** |
| **Rash** |  |  |  |  |  |  |
| No | 74 (86.1) | 25 (89.3) |  | 1 |  |  |
| Yes | 12 (13.9) | 3 (10.7) |  | 0.74 | [0.2-2.8] | 0.66 |
| **Neutrophils (x 10^9^/L)** |  |  |  |  |  |  |
| > 2 | 38 (44.2) | 7 (25.0) |  | 1 |  |  |
| ≤ 2 | 48 (55.8) | 21 (75.0) |  | 2.37 | [0.9-6.2] | 0.076 |
| **Lymphocytes (x 10^9^/L)** |  |  |  |  |  |  |
| > 2 | 13 (15.1) | 2 (7.1) |  | 1 |  |  |
| ≤ 2 | 73 (84.9) | 26 (92.9) |  | 2.31 | [0.5-11] | 0.29 |
| **Monocytes (x 10^9^/L)** |  |  |  |  |  |  |
| > 0.2 | 55 (64.0) | 5 (17.9) |  | 1 |  |  |
| ≤ 0.2 | 31 (36.0) | 23 (82.1) |  | 8.16 | [2.8-23] | **<0.0001** |
| **ASAT^a^ (UI/L)** |  |  |  |  |  |  |
| ≤ 30 | 41 (47.7) | 19 (68.0) |  | 1 |  |  |
| > 30 | 45 (52.3) | 9 (32.0) |  | 0.43 | [0.2-1.1] | 0.067 |
| **ALAT^b^ (UI/L)** |  |  |  |  |  |  |
| ≤ 35 | 53 (61.6) | 25 (89.3) |  | 1 |  |  |
| > 35 | 33 (38.4) | 3 (10.7) |  | 0.19 | [0.05-0.7] | **0.01** |
| **Bilirubin (µmol/L)** |  |  |  |  |  |  |
| ≤ 17 | 76 (88.4) | 22 (78.6) |  | 1 |  |  |
| > 17 | 10 (11.6) | 6 (21.4) |  | 2.07 | [0.7-6.3] | 0.2 |
| **DENV type** |  |  |  |  |  |  |
| DENV-2 | 20 (23.3) | 15 (53.6) |  | 1 |  |  |
| Other DENV | 61 (70.9) | 10 (35.7) |  | 0.22 | [0.1-0.6] | **0.002** |
| Missing data | 5 (5.8) | 3 (10.7) |  |  |  |  |
| **IgG at visit 1** |  |  |  |  |  |  |
| Negative | 45 (52.3) | 15 (53.6) |  | 1 |  |  |
| Positive | 39 (45.4) | 12 (42.8) |  | 0.92 | [0.4-2.2] | 0.86 |
| Missing data | 2 (2.3) | 1 (3.6) |  |  |  |  |
| **NS1 antigen** |  |  |  |  |  |  |
| Negative | 20 (23.3) | 2 (7.1) |  | 1 |  |  |
| Positive | 66 (76.7) | 26 (92.9) |  | 3.94 | [0.8-18] | 0.08 |

^a^ ASAT: Aspartate amino transferase. ^b^ ALAT: Alanine amino transferase.
