# Supplementary material for: Clinical and Virological Study of Dengue Cases and the Members of Their Households: The Multinational DENFRAME Project
Source: PLoS Negl Trop Dis. 2012 Jan 24;6(1):e1482. doi: 10.1371/journal.pntd.0001482 (PMC3265457; doi:10.1371/journal.pntd.0001482)
Supplement: Table S2 — Continuous biological markers observed among non-dengue-infected, inapparent dengue infection and symptomatic dengue-infected subjects. (DOC) [file pntd.0001482.s003.doc]

**Table S2. Continuous biological markers observed among non-dengue-infected, inapparent dengue infection and symptomatic dengue-infected subjects.**

|  |  | Non-dengue-infected (n = 307) | | | |  | Inapparent dengue infection (n = 29) | | | |  | Symptomatic dengue-infected (n = 192) | | | |
| --- | --- | --- | --- | --- | --- | --- | --- | --- | --- | --- | --- | --- | --- | --- | --- |
|  |  | **Mean** | Q1 | Median | Q3 |  | **Mean** | Q1 | Median | Q3 |  | **Mean** | Q1 | Median | Q3 |
| Hematocrit (%) |  | **39.1** | 35.3 | 38.9 | 42.0 |  | **37.0** | 36.0 | 40.0 | 42.0 |  | **40.0** | 37.0 | 40.0 | 43.0 |
| Platelets (x 10^9^/L) |  | **233** | 189 | 222 | 278 |  | **224** | 177 | 234 | 263 |  | **139** | 76 | 137 | 194 |
| Neutrophils (x 10^9^/L) |  | **4.3** | 3.1 | 3.9 | 5.2 |  | **2.7** | 1.6 | 2.4 | 3.0 |  | **2.0** | 1.2 | 1.7 | 2.5 |
| Lymphocytes (x 10^9^/L) |  | **2.7** | 2.1 | 2.6 | 3.2 |  | **2.0** | 1.5 | 2.1 | 2.4 |  | **1.1** | 0.6 | 0.9 | 1.4 |
| Monocytes (x 10^9^/L) |  | **0.4** | 0.3 | 0.4 | 0.5 |  | **0.4** | 0.3 | 0.4 | 0.5 |  | **0.3** | 0.1 | 0.2 | 0.4 |
| ASAT^a^ (UI/L) |  | **25** | 17 | 23 | 31 |  | **29** | 20 | 28 | 38 |  | **37** | 21 | 32 | 49 |
| ALAT^b^ (UI/L) |  | **19** | 13 | 22 | 27 |  | **26** | 13 | 20 | 34 |  | **49** | 17 | 29 | 51 |
| Bilirubin (µmol/L) |  | **13** | 7 | 12 | 17 |  | **11** | 5 | 10 | 17 |  | **13** | 5 | 8 | 15 |

Q1: 25^th^ percentile; Q3: 75^th^ percentile. ^a^ ASAT: Aspartate amino transferase. ^b^ ALAT: Alanine amino transferase.
