# Supplementary material for: Clinical and Virological Study of Dengue Cases and the Members of Their Households: The Multinational DENFRAME Project
Source: PLoS Negl Trop Dis. 2012 Jan 24;6(1):e1482. doi: 10.1371/journal.pntd.0001482 (PMC3265457; doi:10.1371/journal.pntd.0001482)
Supplement: Table S3 — Main characteristics of subjects with acute dengue infection compared to non-dengue-infected subjects. (DOC) [file pntd.0001482.s004.doc]

**Table S3. Main characteristics of subjects with acute dengue infection compared to non-dengue-infected subjects.** Acute dengue-infected subjects belong to dengue index cases (DIC) and household members (HHM). Non-dengue-infected subjects belong to HHM. Univariate and multivariable logistic regression were used for analyses.

|  | Non-dengue-infected  n = 307 (%) | Acute dengue-infected  n = 221 (%) | Crude OR | 95% CI | P* | Adjusted OR | 95% CI | P |
| --- | --- | --- | --- | --- | --- | --- | --- | --- |
| **Sex** |  |  |  |  |  |  |  |  |
| Male | 135 (44.0) | 119 (53.8) | 1 |  |  | 1 |  |  |
| Female | 172 (56.0) | 102 (46.2) | 0.67 | [0.5-0.9] | 0.025 | 0.79 | [0.4-1.5] | 0.48 |
| **Age (years)** |  |  |  |  |  |  |  |  |
| [2 - 7] | 16 (5.2) | 43 (19.5) | 1 |  |  | 1 |  |  |
| ]7 - 10] | 17 (5.5) | 29 (13.1) | 0.63 | [0.3-1.5] | 0.28 | 0.34 | [0.1-1.5] | 0.16 |
| > 10 | 274 (89.3) | 149 (67.4) | 0.2 | [0.1-0.4] | <0.0001 | 0.12 | [0.03-0.4] | **0.001** |
| **Weight-based Z-score** |  |  |  |  |  |  |  |  |
| [-1, 1] | 89 (29.0) | 81 (36.6) | 1 |  |  | 1 |  |  |
| < -1 | 195 (63.5) | 123 (55.7) | 0.69 | [0.5-1.0] | 0.056 | 0.8 | [0.4-1.6] | 0.55 |
| > 1 | 23 (7.5) | 17 (7.7) | 0.81 | [0.4-1.6] | 0.56 | 0.35 | [0.1-1.2] | 0.11 |
| **Hematocrit (%)** |  |  |  |  |  |  |  |  |
| ≤ 36 | 93 (30.3) | 45 (20.4) | 1 |  |  | 1 |  |  |
| > 36 | 212 (69.1) | 176 (79.6) | 1.72 | [1.1-2.6] | 0.01 | 2.34 | [0.9-5.6] | **0.05** |
| Missing data | 2 (0.6) | - |  |  |  |  |  |  |
| **Platelets (x 10^9^/L)** |  |  |  |  |  |  |  |  |
| > 100 | 296 (96.4) | 152 (68.8) | 1 |  |  | 1 |  |  |
| ≤ 100 | 10 (3.3) | 69 (31.2) | 13.4 | [6.7-27] | <0.0001 | 3.35 | [0.9-12] | 0.06 |
| Missing data | 1 (0.3) | - |  |  |  |  |  |  |
| **Neutrophils (x 10^9^/L)** |  |  |  |  |  |  |  |  |
| > 2 | 288 (93.8) | 94 (42.5) | 1 |  |  | 1 |  |  |
| ≤ 2 | 18 (5.9) | 127 (57.5) | 21.6 | [12.5-37] | <0.0001 | 8.08 | [3.6-18] | **<0.0001** |
| Missing data | 1 (0.3) | - |  |  |  |  |  |  |
| **Lymphocytes (x 10^9^/L)** |  |  |  |  |  |  |  |  |
| > 2 | 243 (79.2) | 31 (14.0) | 1 |  |  | 1 |  |  |
| ≤ 2 | 63 (20.5) | 190 (86.0) | 23.6 | [14.8-38] | <0.0001 | 15.2 | [6.7-34.6] | **<0.0001** |
| Missing data | 1 (0.3) | - |  |  |  |  |  |  |
| **Monocytes (x 10^9^/L)** |  |  |  |  |  |  |  |  |
| > 0.2 | 298 (97.1) | 137 (62.0) | 1 |  |  | 1 |  |  |
| ≤ 0.2 | 8 (2.6) | 84 (38.0) | 22.8 | [11-48.5] | <0.0001 | 9.23 | [3.1-28] | **<0.0001** |
| Missing data | 1 (0.3) | - |  |  |  |  |  |  |
| **ASAT^a^ (UI/L)** |  |  |  |  |  |  |  |  |
| ≤ 30 | 225 (73.3) | 92 (41.6) | 1 |  |  | 1 |  |  |
| > 30 | 81 (26.4) | 128 (57.9) | 3.86 | [2.7-5.6] | <0.0001 | 2.9 | [1.4-6.1] | **0.004** |
| Missing data | 1 (0.3) | 1 (0.5) |  |  |  |  |  |  |
| **ALAT^b^ (UI/L)** |  |  |  |  |  |  |  |  |
| ≤ 35 | 261 (85.0) | 134 (60.6) | 1 |  |  | 1 |  |  |
| > 35 | 45 (14.7) | 86 (38.9) | 3.72 | [2.5-5.6] | <0.0001 | 1.68 | [0.8-3.5] | 0.20 |
| Missing data | 1 (0.3) | 1 (0.5) |  |  |  |  |  |  |
| **Bilirubin (µmol/L)** |  |  |  |  |  |  |  |  |
| ≤ 17 | 262 (85.3) | 199 (90.0) | 1 |  |  | 1 |  |  |
| > 17 | 42 (13.7) | 17 (7.7) | 0.53 | [0.3-0.9] | 0.037 | 0.42 | [0.1-1.3] | 0.14 |
| Missing data | 3 (1.0) | 5 (2.3) |  |  |  |  |  |  |
| **IgG at Visit 1** |  |  |  |  |  |  |  |  |
| Negative | 66 (21.5) | 82 (37.1) | 1 |  |  | 1 |  |  |
| Positive | 239 (77.8) | 136 (61.5) | 0.46 | [0.3-0.7] | <0.0001 | 0.44 | [0.2-1] | **0.04** |
| Missing data | 2 (0.7) | 3 (1.4) |  |  |  |  |  |  |

*Potential confounders with a P value of less than 0.20 in univariate analysis were retained for the final multivariable analyses. In this table all variables presented: sexe, age, weight-based Z-score, hematocrit, platelets, neutrophils, lymphocytes, monocytes, ASAT, ALAT, bilirubin and IgG at Visit 1. ^a^ ASAT: Aspartate amino transferase. ^b^ ALAT: Alanine amino transferase.
